# Clinical Effects of Chinese Herbal Decoction Combined with Basic Chemoradiotherapy and Nursing Intervention in the Treatment of Cervical Cancer and the Effect on Serum CEA, CA125, and TNF-*α* Levels

**DOI:** 10.1155/2021/1446864

**Published:** 2021-09-23

**Authors:** Lizhen Gao, Jia Lv, Linlin Hou, Yuchao Yuan, Qiuhua Wan

**Affiliations:** ^1^Department of Clinical Laboratory, Zhangqiu District People's Hospital, Jinan 250200, China; ^2^Department of Obstetrics and Gynecology, Qingdao Hospital of Traditional Chinese Medicine, Qingdao Hiser Hospital, Qingdao 266033, China; ^3^Department of Obstetrics and Gynecology Clinic, Jiyang People's Hospital, Jinan 251400, China; ^4^Department of Obstetrics and Gynecology, Zhangqiu District People's Hospital, Jinan 250200, China; ^5^Department of Clinical Laboratory, Jining Maternal and Child Health and Family Planning Service Center, Jining 272100, China

## Abstract

**Objective:**

This study was aimed to investigate the clinical effect of Chinese herbal decoction combined with basic chemoradiotherapy and nursing intervention in the treatment of cervical cancer and the effect on serum carbohydrate antigen 125 (CA125), carcinoembryonic antigen (CEA), and tumor necrosis factor-*α* (TNF-*α*) levels.

**Methods:**

A total of 200 cervical cancer patients in our hospital from June 2015 to November 2018 were selected and randomly divided into a study group and a control group. The control group was given chemoradiotherapy and psychological nursing treatment, and the study group was given self-made Chinese herbal decoction on the basis of the control group. The clinical efficacy and serum CEA, CA125, and TNF-*α* levels were assessed.

**Results:**

After treatment, the total effective rate of the study group was significantly higher than that of the control group. The levels of serum CEA, CA125, and TNF-*α* were decreased in the two groups after treatment, and the decrease in the study group was more significant than that in the control group. After treatment, CD3^+^ and CD4^+^ levels were increased compared with those before treatment, and the increase in the study group was also more obvious than that of the control group. The level of CD8^+^ was decreased compared with before treatment, and the decrease in the study group was more notable than that of the control group. The two-year cumulative survival rate of the study group was markedly higher than that of the control group. The quality-of-life of patients treated for 3 months, 1 year, and 2 years was dramatically improved compared to before treatment. The incidence of adverse reactions in the study group was lower than that of the control group.

**Conclusion:**

The treatment of basic chemoradiotherapy and psychological nursing intervention combined with Chinese herbal decoction on cervical cancer patients can improve the clinical treatment effects, improve the patient's body immunity, reduce serum CEA, CA125, and TNF-*α* levels, prolong survival time, improve life quality, and reduce the incidence of adverse reactions, and it is worthy of clinical promotion.

## 1. Introduction

Cervical cancer, which originates in the cervix of women, is a common clinical gynecological malignant tumor and currently accounts for the second highest incidence of gynecological malignancies [1]. Its early symptoms are vaginal bleeding and increased leucorrhea, but there is no obvious specificity, and it is not easy to attract patients' attention, thus leading to the majority of patients often having developed to the middle or late stages when diagnosed [2]. Modern medical treatment methods for cervical cancer are surgery, radiotherapy, chemotherapy, immune gene therapy, and so on [3–5]. However, the 5-year survival rate of patients after treatment is extremely low, indicating that it will have a certain negative impact on patients and seriously affect the life and health of patients [6]. Rogers et al. pointed out that chemoradiotherapy can be used to treat cervical cancer, which can kill tumor cells better and prolong the survival time of patients [7]. However, chemoradiotherapy will aggravate the adverse reactions of patients after treatment, and patients have poor tolerance to treatment, and the treatment effect is not ideal. Li et al. suggested that the combination of Chinese herbal decoctions and conventional treatment can effectively improve the immune function of patients, thereby reducing the toxic side effects of the treatment on patients [8]. With the development of the traditional Chinese medicine (TCM) industry in China, TCM has a unique advantage in treating cervical cancer and other gynecological tumors because of its characteristic of syndrome differentiation and rich experience in gynecological treatment [9]. However, the wide variety of TCM and the different compatibility methods make the current analysis of the cervical cancer prescriptions limited to the summary of the experience of famous doctors [10]. Therefore, this study explores the clinical effects of Chinese herbal decoction combined with basic chemoradiotherapy and nursing intervention on cervical cancer and the effects on CEA, CA125, and TNF-*α* levels. The report is as follows.

## 2. Materials and Methods

### 2.1. General Information

A total of 200 cervical cancer patients admitted at Zhangqiu District People's Hospital, Jinan, China, from June 2015 to November 2018 were included in the study and randomly divided into a study group and a control group, with 100 cases each. Inclusion criteria: (1) Western medicine diagnoses, all meet the relevant diagnostic criteria according to the “International Union of Obstetrics and Gynecology 2015 Guidelines for the Diagnosis and Treatment of Cervical Cancer” [11], (2) all patients diagnosed by pathological biopsy, (3) Chinese medicine diagnoses, all meet the diagnostic criteria of the “Guiding Principles for Clinical Research of New Chinese Medicines” [12], (4) all patients are the first treatment, (5) the expected survival period is ≥ 6 months, (6) patients with complete clinical data, and (7) all patients provided written informed consent. Exclusion criteria: (1) patients with liver and kidney dysfunction, (2) patients with other malignant tumors, (3) patients who are allergic to the drugs in this study, and (4) patients who are difficult to complete the study. This study was approved by the ethics committee of the Zhangqiu District People's Hospital, Jinan, China, and conducted in accordance with the Declaration of Helsinki. There was no statistically significant difference in general information between the two groups (Table 1) (*P* > 0.05), which was comparable.

### 2.2. Treatment Method

The control group was treated with chemoradiotherapy. Cisplatin injection (30 mg/m^2^, H20010743, Howson Pharmaceutical Group Co., Ltd., Jiangsu) was diluted with 250 ml of physiological saline, intravenous drip, 1 time/week, for total treatment 12 weeks. On this basis, the study group was given combined treatment with self-made Chinese herbal decoction. The composition of the soup is as follows: *Scutellaria barbata* (30 g), *Coix* seed (30 g), *Atractylodes macrocephala* (12 g), *Oldenlandia diffusa* (30 g), *Codonopsis pilosula* (9 g), *Poria cocos* (15 g), *Astragalus* (30 g), *Angelica* (9 g), Radix Paeoniae Alba (9 g), *Bupleurum* (9 g), and *Cyperus tuber* (9 g). Decoction method: all above medicine was put in a medicine pot with clear water, preferably 3 cm under the medicine, soaked for 30 minutes, and decocted 2 times to 300 ml concoction. Chinese herbal decoction started to be taken on the first day of chemotherapy, 1 dose/day, warm clothing in the morning and evening, 4 weeks as a course of treatment, a total of 3 courses of treatment. All patients were given certain psychological counseling to eliminate their worries. The patients will feel fear and anxiety, especially when the lower body feels pain or bleeding. The benefits and effects of TCM treatment should be introduced to patients to increase their confidence.

### 2.3. Detection Methods of Serum Markers

The serum tumor marker levels were detected one day before treatment and at the end of 3 treatment courses of the patients. 5 ml of fasting venous blood was extracted from the patient. After standing for 1 h, the blood was centrifuged at 3000 r/min for 10 min. The supernatant was drawn into the EP tube using a pipette. The enzyme-linked immunosorbent assay (ELISA) was performed to detect CA125, CEA, and TNF-*α* by the ARCHITECT i4000SR automatic immunoassay analyzer (Abbott, USA) and special kits according to the instructions.

### 2.4. Observation Index

The clinical efficacy of patients after treatment was evaluated, the evaluation criteria are complete remission (CR, the lesions completely subsided for more than one month), partial remission (PR, the lesion has shrunk >50% for more than one month, and there is no trend of new lesions appearing, growing, or becoming serious), and uncontrolled (NC, the enlargement or reduction of the lesion is less than 50%, or discovery of the emergence, growth, and severity of new lesions) [13]. Total effective rate = (CR + PR)/total number × 100%. After 3 months of treatment, patients were reexamined to observe the immunological status of patients before and after treatment. Immunoglobulin tests were performed one day before treatment and after treatment. 5 ml of fasting venous blood was drawn from the patient. The test instrument was a flow cytometer (CyFlow® Cube 8, Partec, Germany). The levels of T lymphocyte subsets in peripheral blood including CD3^+^, CD4^+^, and CD8^+,^ were detected by fluorescent molecular labeling. Serum tumor markers were observed before and after treatment. The adverse drug reactions in 2 groups were recorded, including bone marrow suppression, alopecia, radioactive rectitis, cardiac function damage, gastrointestinal side effects, radioactive dermatitis, and renal dysfunction. We followed up the patients within 2 years after the end of treatment, evaluated the two-year survival rate, and conducted a quality-of-life survey (FACT-G). Before treatment, 3 months after treatment, 1 year after treatment, and 2 years after treatment, professional scorers were assessed according to the patients' true responses. The table includes four major items: physiological status, social/family status, emotional status, and functional status, and the total score is calculated. The lower the score, the worse the quality-of-life is affected by the disease.

### 2.5. Statistical Methods

SPSS 22.0 software was used for the statistical analysis. Measurement data are expressed as mean ± standard deviation ($\bar{x}\pm s$), count data are expressed as *n* (%), and the comparison used the *χ*^2^ test. The difference is statistically significant with *P* < 0.05.

## 3. Results

### 3.1. Comparison of the Clinical Efficacy of the Two Groups Patients

The total effective rate of treatment for patients in the study group was 81.00%, and it was 58.00% in the control group (Table 2). The clinical efficacy of patients in the study group was significantly higher than that of the control group (*χ*^2^ = 13.039, *P*=0.001) (Table 2).

Comparison of serum CEA, CA125, and TNF-*α* levels between the two groups.

**Figure 1:**
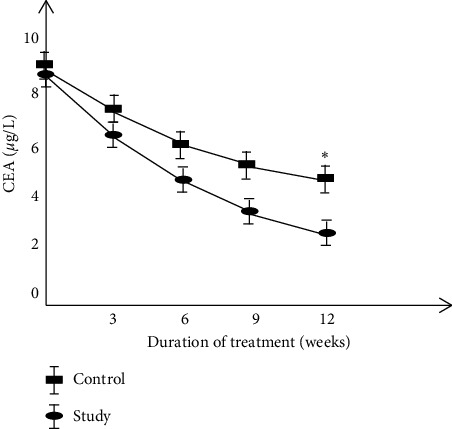
Comparison of serum CEA levels between two groups.

**Figure 2:**
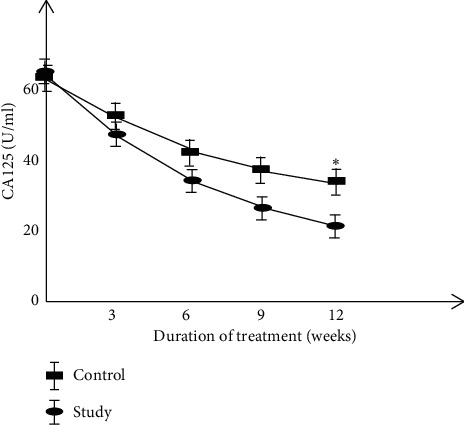
Comparison of serum CA125 levels between two groups.

**Figure 3:**
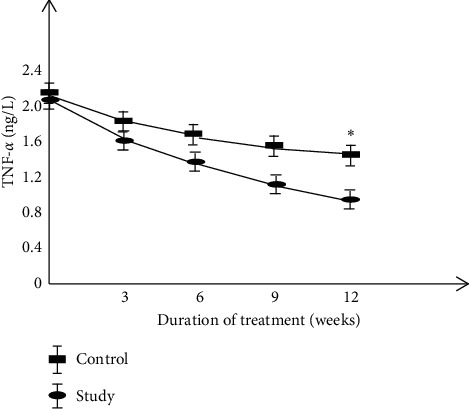
Comparison of serum TNF-*α* levels between two groups.

Before treatment, there was no significant difference in the levels of serum CEA, CA125, and TNF-*α* between the two groups of patients. The levels of serum CEA, CA125, and TNF-*α* were decreased after treatment in the two groups, and the study group decreased more significantly than the control group (*P* < 0.05) (Figures 1–3 ).

### 3.2. Comparison of Serum Immune Function between Two Groups

**Figure 4:**
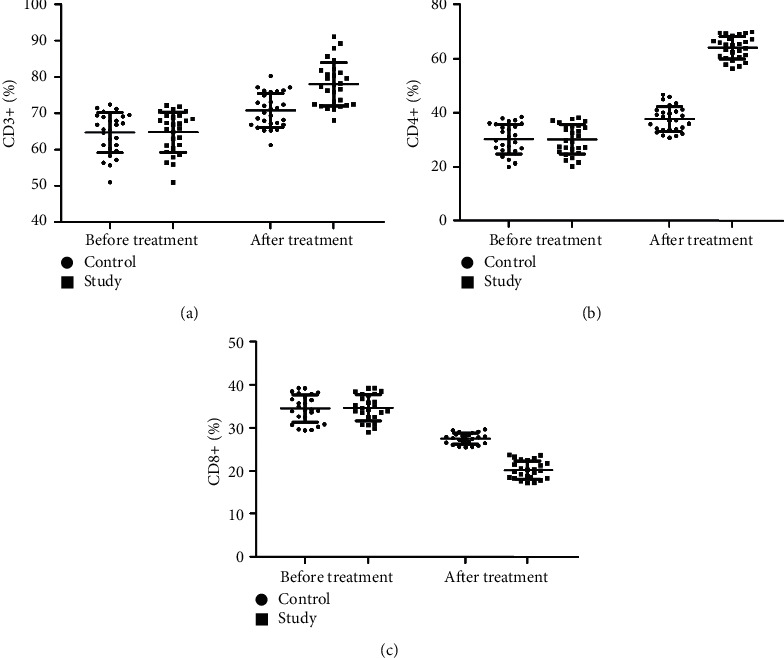
Comparison of CD3^+^, CD4^+^, and CD8^+^ levels between the two groups.

There was no significant difference in CD3^+^, CD4^+^, and CD8^+^ levels between the two groups before treatment. CD3^+^ and CD4^+^ levels were increased after treatment in two groups, and the increase in the study group was more significant than that in the control group (*P* < 0.05) (Figures 4(a) and 4(b)). CD8^+^ levels were decreased compared with those before treatment, and the decrease in the study group was more significant than that in the control group (*P* < 0.05) (Figure 4(c)).

### 3.3. Two-Year Cumulative Survival Rate and Quality-of-Life Were Compared between the Two Groups

**Figure 5:**
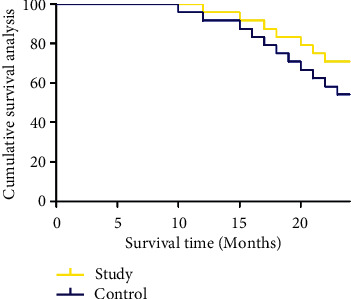
Comparison of two-year cumulative survival rate between the two groups.

The two-year cumulative survival rate was significantly higher in the study group (70.83%) than in the control group (54.17%) (*P* < 0.05) (Figure 5). There was no significant difference in the quality-of-life between the two groups before treatment (*P* > 0.05). However, for 3 months after treatment, 1 year after treatment, and 2 years after treatment, the quality-of-life was significantly improved (*P* < 0.05) (Table 3).

### 3.4. Comparison of the Incidence of Adverse Reactions between the Two Groups of Patients

The incidence of adverse reactions in the study group was 18% and 47% in the control group (Table 4). The difference was statistically significant (*χ*^2^ = 12.664, *P*=0.001) (Table 4).

## 4. Discussion

Cervical cancer with high morbidity and mortality is a common malignant tumor that threatens the health and life safety of women [14]. The symptoms of early cervical cancer are concealed, and there are no specific clinical signs, which are easy to be ignored by patients. When clinical symptoms appear, they are often in the middle and late stages, missing the right for surgery [15]. Additionally, patients with advanced cervical cancer are often accompanied by cancer cell dissemination or lymph node metastasis, thus leading to radical surgery that cannot be carried out [16]. At present, chemoradiotherapy is often selected as the main treatment to improve the survival rate of patients [17]. Chemoradiotherapy for cervical cancer can shrink tumors and enhance parauterine invasion [18]. Chemotherapy inhibits the reproliferation of tumor cells during the interval of radiotherapy and the repair of sublethal tumor cell damage after radiotherapy, synchronizes the tumor cell cycle, inhibits the repopulation of tumor cells, improves the sensitivity of tumors to radiotherapy, and reduces the recurrence rate, thus improving the prognosis [19–21]. The standard regimen of chemoradiotherapy is often based on cisplatin [22–25]. Cisplatin is one of the most effective drugs for the clinical treatment of metastatic cervical cancer [22]. Its response rate is as high as 50%. It can inhibit DNA replication and transcription, leading to DNA breaks and code errors. However, most patients with advanced cervical cancer have a certain degree of decline in immune function. Cisplatin synchronous radiotherapy will bring some toxic side effects to patients, such as nausea, vomiting, and bone marrow suppression, which further reduces the patient's immune function and affects the treatment effect, increases the patient's suffering, and further affects the patient's quality-of-life and treatment compliance. The psychological nursing intervention during chemoradiotherapy can improve the prognosis of patients and effectively reduce the incidence of complications. Research data prove that psychological care can make patients psychologically happy, effectively reduce the degree of unhappiness, patients can be in the best physical and mental state to cooperate with clinical treatment work, effectively control the incidence of complications, and accelerate the patient's early recovery process [26, 27]. While reducing the incidence of complications, patient care satisfaction increases.

TCM believes that cervical cancer belongs to the categories of “abdominal mass,” “uterine bleeding,” and “multicolored leucorrhea” [28]. The leading cause of the disease is menstruation, postpartum wind-cold-damp pathogen, and other evil poison invasions, coupled with the diet, seven emotions disorder, and other factors, resulting in weakness of the spleen and stomach, liver and kidney loss, deficiency of qi and blood, and damage the Chong channel and Ren channel [29]. When there is a problem with the Chong channel and Ren channel, the Du channel becomes weak, and the Dai channel is unstable, resulting in morbid vaginal discharge, and the blood stasis and damp toxin accumulate in the lower abdomen and the uterus to form a mass [30]. For a long time, evil poison is in depth, so that the healthy qi deficiency loss and internal and external evils result in qi stagnation and blood stasis and cancer toxin build-up [30]. In addition, Chinese medicine experts believes that chemotherapy drugs are toxic substances, which often further damage qi and blood, deplete qi and injure yin, and further damage the body's internal organs, causing spleen and kidney yang deficiency, spleen and stomach deficiency and cold, and stomach disharmony, insufficient qi and blood [31]. For cervical cancer, experts in Chinese medicine have confidence that treatment should be based on the theory of the spleen, stomach, and kidney, which can also lessen the adverse reactions caused by chemotherapy in patients to some extent [32]. Therefore, the clinical treatment should be based on strengthening vital qi to eliminate pathogenic factors, resolving toxins to transform stagnation and blood-enriching and supplementing qi. The basic prescription used by patients in this study included *Astragalus* and *Codonopsis pilosula* as the monarch drugs, which could invigorate spleen-stomach and replenish qi and raise yang and lift collapse [33]; *Atractylodes macrocephala* can tonify kidney essence, invigorate the spleen and kidney, effectively correct qi deficiency and weakness, and middle qi collapse [33]; *Scutellaria barbata* and *Coix* seed can clear heat and remove toxicity [33]; white Radix Paeoniae Alba and *Cyperus tuber* can enrich blood qi [33]; *Poria cocos* can nourish the spleen and disinhibit dampness, eliminate dampness, and inhibit tumor [33]; *Oldenlandia diffusa* can clear heat and dissipate phlegm [33]; *Angelica* can not only invigorate blood but also activate blood, can not only dredge the meridian but also activate collaterals, and has the effect of pain-killing and anticancer [34]. The whole prescription is compatible with both symptoms and root causes, with the main function of improving vital qi, supplemented by the elimination of evils, and it has the effects of strengthening the vitality and replenishing qi, detoxifying and removing blood stasis, dredging the meridian and eliminating symptoms, and anticancer and tumor suppressing.

The results of this study showed that the clinical efficacy of the study group after treatment was notably higher than that of the control group, and immune function was significantly increased while the incidence of adverse reactions was decreased. These results indicated that basic therapy combined with TCM in the treatment of cervical cancer could reduce the toxic and side effects of patients and improve the treatment tolerance of patients. Molecular immunology believes that the basis of maintaining the body's cellular immune function is T cell subsets [35]. The immune function of patients with cervical cancer is low, which makes the body's T cell subsets disorder, and the expression of each index is also abnormal. After treatment, the level of the patient's T cell subsets improved better, and the clinical symptoms were controlled. The results of this study showed that the two-year cumulative survival rate and improvement rate in quality-of-life after treatment were significantly higher in the study group than those of the control group. It is suggested that compared with cisplatin simultaneous radiotherapy treatment, combined with self-made Chinese herbal decoction adjuvant treatment can further improve the clinical efficacy of cervical cancer patients, enhance the body's immune function, thereby reducing the occurrence of serious drug toxicity, and exerting a good effect of increasing efficacy and reducing toxicity. The serum levels of CEA, CA125, and TNF-*α* in the study group were decreased more notably than those in the control group, which is consistent with the results of Huang et al. and Li et al. [36, 37], suggesting that the Chinese herbal formula has a better anti-tumor effect and can improve the abnormal expression of tumor markers.

## 5. Conclusion

In conclusion, certain psychological nursing interventions and chemoradiotherapy combined with traditional Chinese medicine decoction treatment for cervical cancer patients have good therapeutic effects, at the same time, improve the patient's serum tumor markers and immune level, and the treatment method is highly feasible.

## Figures and Tables

**Table 1:** Comparison of general clinical data of the two groups of patients (*n*).

Index	Study	Control	*X* ^2^	*P*
Cases	100	100		
Age (year)			0.082	0.774
>55	57	59		
≤55	43	41		
BMI (kg/m^2^)			1.456	0.483
<18.5	13	15		
18.5–24.9	69	73		
≥25	18	12		
Course (year)			0.357	0.550
2–4	32	36		
<2	68	64		
Pathological type			0.231	0.891
Squamous cell carcinoma	56	58		
Adenocarcinoma	29	26		
Adenosquamous carcinoma	15	16		
Clinical stage			0.245	0.885
I	26	23		
II	44	46		
III	30	31		

**Table 2:** Comparison of clinical efficacy between the two groups of patients.

Group	Cases	CR	PR	NC	Total effective rate (*n* (%))
Study	100	43	38	19	81.00%
Control	100	27	31	42	58.00%
*χ* ^2^					13.039
*P*					0.001

**Table 3:** Comparison of quality-of-life between the two groups.

Group	Before treatment	3 months after treatment	1 year after treatment	2 years after treatment
Study	51.43 ± 5.66	73.83 ± 7.92	95.46 ± 4.21	105.62 ± 4.67
Control	52.14 ± 5.71	62.34 ± 6.58	74.72 ± 6.11	81.73 ± 5.85
*χ* ^2^	0.623	7.733	11.851	13.984
*P*	0.482	0.001	≤0.001	≤0.001

**Table 4:** Comparison of the incidence of adverse reactions between the two groups of patients (*n*).

Group	Cases	Bone marrow suppression	Alopecia	Radioactive rectitis	Cardiac function damage	Gastrointestinal side effects	Radioactive dermatitis	Renal dysfunction
Study	100	2	7	2	1	3	1	2
Control	100	6	12	7	5	7	4	6

## Data Availability

The data generated or analyzed during this study are included within this article.
